# The use of a protein network analysis to explore the complexity of early skin inflammation after oronasal mask application- A pilot study

**DOI:** 10.1038/s41598-024-67583-9

**Published:** 2024-07-19

**Authors:** Amanda Feldt, Bijar Ghafouri, Peter R. Worsley, Sara Bergstrand

**Affiliations:** 1 Department of Acute Internal Medicine and Geriatrics in Linköping, and Department of Health, Medicine and Caring Sciences, Linköping University, 581 83 Linköping, SE Sweden; 2 Pain and Rehabilitation Centre, and Department of Health, Medicine and Caring Sciences, Linköping University, Linköping, Sweden; 3 Skin Sensing Research Group, School of Health Sciences, University of Southampton, Southampton, UK; 4 Department of Health, Medicine and Caring Sciences, Linköping University, Linköping, Sweden

**Keywords:** Chemokines, Cytokines, Inflammation, Predictive markers, Prognostic markers, Protein-protein interaction networks, Molecular medicine, Translational research

## Abstract

Medical devices, such as non-invasive ventilation masks, save lives in health care settings but can be a cause of tissue injuries due to the pressure and shear loads on skin and soft tissue. These pressure injuries could be painful for the individual and cause a significant economic impact on healthcare providers. In the etiology of device related pressure ulcers, inflammation plays an important role. However, the exact nature and timing of inflammatory biomarker upregulation is still unknown in the early stages of skin damage. This study aimed to explore the inflammatory profile of vulnerable skin sites following non-invasive mask application on a convenience sample of eleven hospital patients. Seventy-one inflammatory proteins were explored from sebum sampled at the skin surface after oronasal mask application. A multivariate analysis to investigate differences between loaded and control site was conducted, with a protein network analysis used to explore interactions in the early inflammation. The study revealed that 21 cytokines and chemokines were important for the separation between loaded and control site. These proteins were associated with remodeling of tissue, vascular wound healing and/or cell death.

## Introduction

Pressure ulcers (PUs) are a significant burden for individuals, with studies demonstrating that they decrease quality of life and cause pain^1^, as well as an economic impact on health care settings for wound care^2^. Pressure ulcers could be avoidable, yet it is one of the most prevalent health problems worldwide^3^. One of the causes of PUs are medical devices, which can cause skin damage directly under the device from prolonged pressure and/or shear. The National Pressure Injury Advisory Panel (NPIAP) states that medical device-related pressure ulcers ‘…result from the use of devices designed and applied for diagnostic or therapeutic purposes. The resultant pressure injury generally conforms to the pattern or shape of the device.’ There are many different examples of medical devices which impinge on vulnerable skin sites, often seen in critical care settings^4^.

Despite the respiratory benefits of non-invasive ventilation (NIV) being widely accepted, it is one of the devices implicated in pressure ulcer formation^5^. The specific incidence of NIV-related pressure ulcers has been shown to range from 5 to 50% for 2–4 h of continuous usage and up to 100% after 48 h of wearing a face mask^6^. In a recent meta-analysis, the pooled prevalence of NIV-related pressure ulcers was 25%^7^. NIV is delivered through an oral nasal face mask attached to the individual to manage a range of respiratory disorders. NIV masks cause pressure and shear at the skin interface, which results in tissue deformation which can harm skin and sub-dermal tissues^4^. The face masks affect the tissue in contact as well as the microclimate underneath the mask, which may cause a reduced tolerance to mechanical pressure^8^. NIV mask application leads to increased loading in the chin, cheeks and at the nasal bridge^9,10^. Since the nasal bridge has a small contact area and corresponds to a bony prominence with limited soft tissue coverage, it creates high localized pressure and frictional force, creating a site of high vulnerability^11^. This vulnerability increases the risk of non-blanching erythema that may quickly develop to full thickness tissue injury down to the bone^4^.

Since device-related pressure ulcers seem to develop faster than pressure ulcers from body weight^12^, there is a need to identify early tissue damage. Research has explored methods for objectively detecting early-stage tissue damage^13^, but these have not been translated in the daily work by the health care staff. Further, there are no clinical guidelines of specified NIV mask strapping tension during treatment, or recovery period of skin after treatment which could prevent tissue damage. As a result, there is little standardization in device application and skin health checks to prevent pressure ulcers.

Previous research has explored the inflammatory response from increased loading in skin, using different methods such as tape stripping, bioimaging, and transepidermal water loss^14,15^. Non-invasive assessment of inflammatory biomolecules in sebum released at the skin surface using adhesive tape provides further assessment of the tissue status in skin as the released biomolecules origin in superficial stratum corneum, as well as the deeper dermis and the sebaceous glands^16^. The method has been validated^17^ and subsequently optimized^18^, with emerging evidence exploring the inflammatory cytokines after increased loading in skin^19,20^. Indeed, IL-1α, IL-8 and IL-1Ra have been identified as potential biomarkers for pressure ulcers^21^. However, these studies have been limited by panels offered by standard ELISA techniques, offering a limited scope to explore the range of inflammatory biomarkers involved in the tissue response to mechanical load.

The aim of the present study was to explore the inflammatory profile of vulnerable skin sites following NIV mask application, which would lead to an improved scientific understanding of pressure ulcer formation. To our knowledge, this is the first study to simultaneously explore 71 different inflammatory proteins from mechanically loaded skin and to use a protein network analyse to contribute with increased knowledge in the complexity of early inflammation.

## Material and methods

### Subjects

Eleven patients from a single thoracic intensive care unit in Sweden were recruited to this observational study. The inclusion criteria were admission to thoracic intensive care and the need of non-invasive ventilation treatment with oronasal face masks. The sole exclusion criterion was skin tissue damage at the measurement areas in the face. All included patients received their usual care and treatment prescribed by the physician.

### Ethics

**Figure 1:**
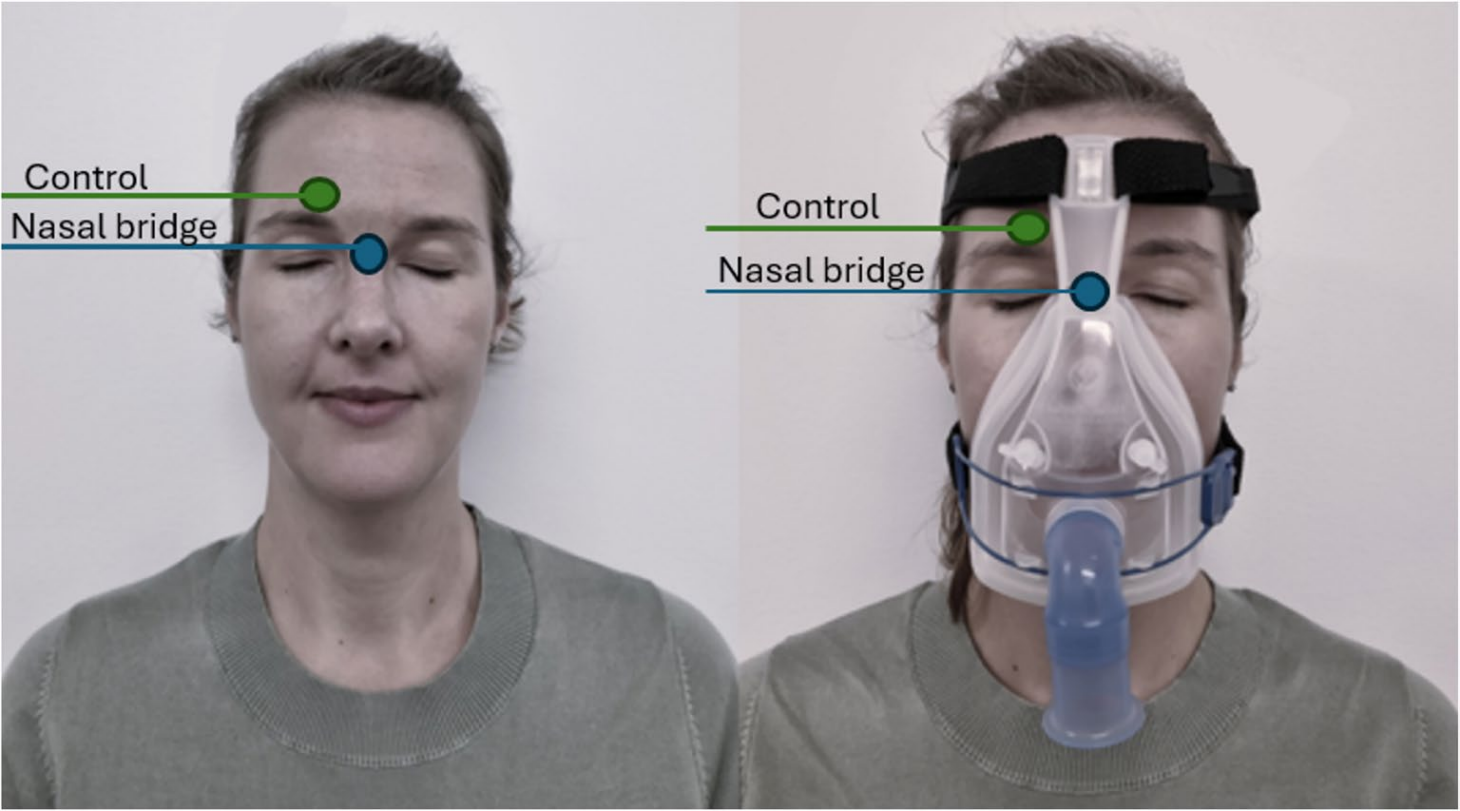
Sampling sites of sebum. The blue dot represents nasal bridge, the green dot represents control site. Sampling using Sebutape before and after NIV-therapy.

The study was approved by The Swedish Ethical Review Authority (Dnr 2018/102-31, 2019-04951). All participants gave their informed consent to participation, or if unable to consent, in consultation with their next of kin. An informed consent was obtained from the person in Fig. 1 to publish the image in an online open access publication. The research has been performed in accordance with the Declaration of Helsinki^22^. Accordingly, The Swedish General Data Protection Regulation (GDPR) and the Patient Data Act (2008:355) were followed to ensure the security of collected and stored personal data and information.

### Data collection

Sebum samples from skin were collected at baseline and directly after NIV-therapy from the nasal bridge under the oral nasal mask and from an unloaded control area in the forehead (Fig. 1) for 2 min using a commercial tape (Sebutape, CuDerm, Dallas, TX, USA), according to validated protocols^17^. The duration of NIV-therapy was included in the data collecting since all patients received individualized treatment. Gloved hands and tweezers were used to minimize the risk of sample contamination. The samples were put in test tubes marked with sample number and the individuals study number. The samples were stored in a freezer at − 86 ℃ until analysis.

### Biochemical analysis

A U-PLEX assay based on an electrochemiluminescent detection method (Meso Scale Diagnostics, Rockville, MD, USA) was used to analyse the concentrations of 71 cytokines and chemokines according to the manufacturer’s recommendations. Data was analysed using MESO QUICKPLEX SQ 120 instrument equipped with data analysis software DISCOVERY WORKBENCH (Meso Scale Diagnostics, Rockville, MD, USA).

The tapes were thawed on the day of the analysis and proteins were extracted by adding 300 µl phosphate (PBS) buffer containing 0.05% tween and mixed gently for 1 h at + 4 ℃. The samples were centrifuged, and the supernatant moved to a new tube. The concentration of cytokines/chemokines were measured using MSD according to the manufacture's manual. Briefly, 96-well plates were coated with linker-coupled capture antibodies (provided by the manufacturer) for one hour, aspirated and washed with washing buffer (PBS/0.05% Tween-20) for three times. Sebum samples (25 µl) were added to wells and incubated for one hour at rooms temperature with shaking. Thereafter the fluids were removed, and the wells were washed three times with washing buffer. Detection antibodies were added to each well, incubated for one hour at rooms temperature and washed three times. Subsequently, 150 µl of reading buffer was added to each well and the plate were immediately analysed on the MSD instrument. Standard curves were formed by fitting electrochemiluminescence signal from calibrators to a weighted 4-parameter logistic model. For the purposes of statistical analyses, any value that was below the lowest limit of detection (LLOD) for the assay was replaced with half of LLOD of the assay.

### Statistical analysis

Descriptive statistics of the clinical data were analysed using SPSS, version 28.0 (IBM Corp.; Armonk, NY, USA). The proteomic data set was analysed by a multivariate data analysis using the software SIMCA (version 17.0; Sartorius Stedim Biotech, Umeå, Sweden) as earlier described^23,24^ and in accordance with Wheelock and Wheelock^25^. Variables were mean centered and scaled for unified variance (UV-Scaling). A principal component analysis (PCA) was performed to check for outliers using score plots and Hotelling`s T2 and distance to model in X-space. An orthogonal partial least square discriminant analysis (OPLS-DA) was used to regress group discrimination, determining which cytokines/chemokines were important for class differences between control/baseline and the vulnerable skin site following increased loading. The variable influence of projection (VIP) was used to measure the importance of each variable. P(corr) represents the loading of each variable scaled as a correlation coefficient. VIP > 1.0 and p(corr) > 0.4 were considered as significant.

Other model diagnosis parameters included the goodness of fit (R^2^), and the goodness of prediction (Q^2^). R^2^ is represented by the fraction of sum of squares of all the variables explained by a principal component. Q^2^ are the fraction of total variation of the variables that can be predicted by a principal component using cross validation methods. The differences between R^2^ and Q^2^, should not be above > 0.3, which implies that the robustness of the model is weak^24^.

Cross validated analysis of variance (CV-ANOVA) was conducted for the validity of the model. A p-value < 0.05 for CV-ANOVA was considered as significant. The Mann-Whitney U test was conducted in SPSS since the data was not normally distributed, to test the significance level of comparison between the potential biomarkers. A p-value < 0.05 was considered as significant.

### Bioinformatics

The bioinformatic tool STRING (Search Tool for Retrieval of interacting Genes/Proteins), version 11.5, was used to explore known and predicted protein–protein interactions^26^. Protein accession numbers were entered in the multiple proteins search engine for all detected significant proteins (VIP > 1). The following settings were used: Organism Homo Sapiens; interaction score was set to high confidence (0.70); the maximum number of interactions were only query proteins and an FDR (false discovery rate) < 0.05 was used when classifying the Biological Process (GO) of each protein. For the protein network, protein–protein interaction (PPI) enrichment p value was reported. In the network figure, protein–protein interaction and association are represented by a line, and each protein is represented as a colored node. Higher combined confidence scores are represented by thicker lines.

## Results

### Overview

Eleven patients, 7 men and 4 women at mean age of 69 ± 6 years participated in the study. Their mean body mass index (BMI) was 29.1 kg/m2 (± 6.2) and they had spent in mean 3 ± 2 days in the intensive care unit. All patients had undergone thoracic surgery, most common procedure was valve replacement (n = 7) and/or coronary artery bypass graft (n = 4), which caused systemic inflammation, CRP mean 120.8 mg/l (± 137.6). All patients were well monitored and were observed to be normal in their vital signs.

All patients underwent their prescribed NIV-treatment with oronasal face mask, the mean duration time was 22 ± 8 min. Eight patients obtained PU preventive gel pads at the nasal bridge during the therapy session. Visible erythema at the nasal bridge after the NIV therapy were seen in 7 patients (64%). In this study, 71 different proteins in sebum were collected. Proteins below the limit of detection in more than 50 percent of the study population were excluded from further analysis (n = 11). There was no missing data.

### Differences in inflammatory proteins between timepoint

**Figure 2:**
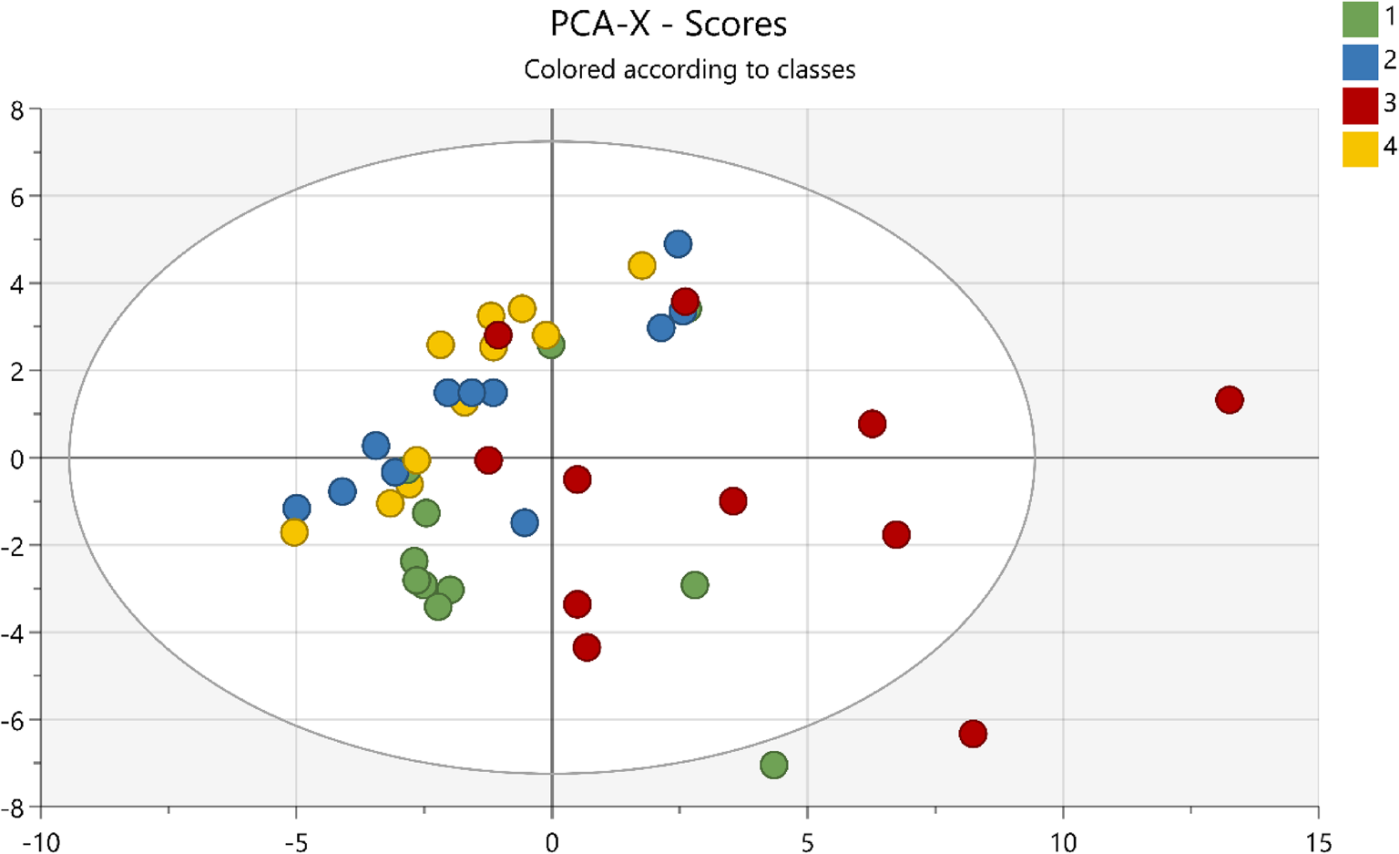
Principal Component Analysis (PCA) of the proteome over time. The score plot shows the changes of protein expression in all measurements points; baseline nasal bridge (green), baseline control (blue), control after NIV (yellow) and nasal bridge after NIV (red). The X-axis represents intergroup differences, and the Y-axis represents intragroup differences.

Multivariate statistical analysis in the remaining 60 proteins that were above the limit of detection was used to investigate differences in protein response before and after NIV therapy. The unsupervised PCA comprised two components, analysis identified one moderate outlier. As shown in Fig. 2, a cluster including samples from control sites (blue circles = baseline and yellow circles = after NIV) and nasal bridge at baseline (green circles) was observed. Thereby the samples from these sites and timepoints are called control samples.

**Figure 3:**
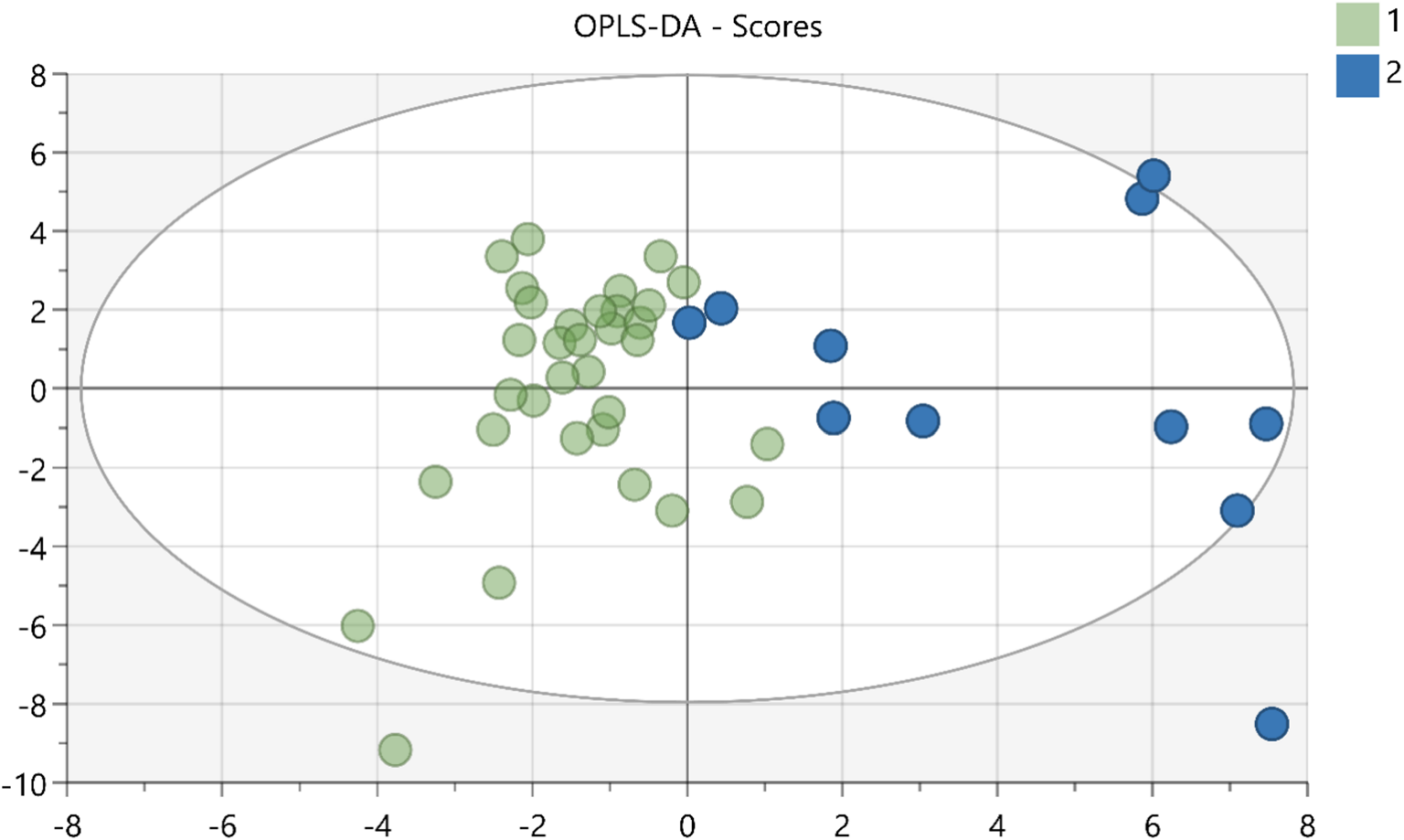
An orthogonal partial least square discriminant analysis (OPLS-DA) of the proteome over time. The score plot shows the changes of protein expression in 60 proteins between two classes: baseline nasal bridge and control area (green) and nasal bridge after NIV (blue). R2 0.68, Q2 0.47, CV ANOVA p-value = < 0.001. The X-axis represents intergroup differences, and the Y-axis represents intragroup differences.

**Figure 4:**
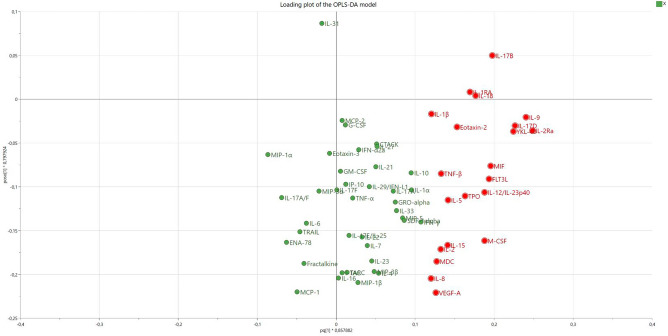
Loading plot of the OPLS-DA-model. The loading plot shows the 60 proteins and are a complement to the score plot illustrated in the previous Fig. 3. Hence, proteins more to the right in Fig. 4, represents the loaded group in the score plot (Fig. 3), which illustrates which proteins that are of interest. The 21 proteins most important for separation between loaded and unloaded groups are marked as red.

The concentration of all 60 proteins from samples from the two groups (controls (n = 33) and directly after NIV (n = 11)) was analysed using an orthogonal partial least square discriminant analysis (OPLS-DA). This model consisted of one predictive and one orthogonal component with sensitivity (R^2^ = 0.68), predictivity (Q^2^ = 0.47), and a significant CV-ANOVA (p-value < 0.001) showing significant differences between sites. Data are illustrated in Fig. 3*.* The loading plot of the OPLS-DA model is visualized in Fig. 4*.*

Following OPLS-DA analysis, 21 proteins were identified to contribute most to the separation, using the threshold VIP > 1 and |*p*(corr)|> 0.4) (Table 1)*.*

**Table 1:** The 21 most important proteins for the OPLS-DA model.

Protein name (short name)	Access-sion number	Control/baseline pg/ml median (min–max)	Nasal bridge pg/ml median (min–max)	LLOD pg/ml	VIP pred	p(corr)	p-value	Biological process
C–C motif chemokine 22, MDC (CCL22)	O00626	17.91 (0.00–48.93)	28.77 (4.84–119.18)	23.10	1.13	0.44	0.069	InfRes, ImmSysPro
C–C motif chemokine 24, Eotaxin-2 (CCL24)	O00175	0.53 (0.00–32.42)	9.23 (2.16–46.07)	6.20	1.36	0.53	0.003*	InfRes, ImmSysPro
Chitinase-3-like protein 1, YKL-40 (CHI3L1)	P36222	3397.24 (944.35–7463.46)	9252.86 (3569.25–13861.07)	0.57	1.99	0.78	0.99	InfRes, ImmSysPro
Fms-related tyrosine kinase 3 ligand (FLT3LG)	P49771	0.00 (0.00–23)	0.09 (0.00–0.31)	0.14	1.72	0.68	0.009*	ImmSysPro, RegCelDet
Interleukin-1 beta (IL1-β)	P01584	5.72 (0.55–51.96)	16.81 (3.53–47.90)	0.06	1.07	0.42	0.013*	InfRes, ImmSysPro, RegCelDet
Interleukin-1 receptor antagonist protein, (IL1Ra)	P18510	3928.99 (582.00–4853.27)	4833.83 (4355.60–5075.77)	1.05	1.50	0.59	< 0.001*	InfRes, ImmSysPro
Interleukin-2 (IL2)	P60568	0.06 (0.00–33)	0.15 (0.00–0.51)	0.23	1.18	0.46	0.53	ImmSysPro, RegTissRem, PosRegTisRem, RegCelDet
Interleukin-2 receptor subunit alpha (IL2Ra)	P01589	0.00 (0.00–16.65)	17.29 (0.00–25.44)	5.15	2.21	0.87	< 0.001*	InfRes, ImmSysPro
Interleukin-5 (IL5)	P05113	0.02 (0.00–14)	0.15 (0.00–0.20)	0.12	1.26	0.5	0.015*	InfRes, ImmSysPro
Interleukin-8 (CXCL8)	P10145	3.70 (0.45–85.25)	8.13 (1.12–132.74)	0.06	1.07	0.42	0.069	InfRes, ImmSysPro
Interleukin-9 (IL9)	P15248	0.00 (0.00–23)	0.22 (0.00–0.37)	0.04	2.13	0.84	< 0.001*	InfRes, ImmSysPro
Interleukin-12 subunit beta, IL-12/IL-23p40 (IL12B)	P29460	0.48 (0.00–2.37)	1.67 (0.00–6.98)	0.77	1.67	0.65	0.009*	ImmSysPro, RegTissRem, PosRegTisRem RegCelDet
Interleukin-15 (IL15)	P40933	0.15 (0.00–54)	0.24 (0.01–0.79)	0.30	1.25	0.49	0.009*	InfRes, ImmSysPro, RegTissRem, PosRegTisRem
Interleukin-17B (IL17B)	Q9UHF5	0.00 (0.00–78)	0.66 (0.00–2.29)	0.46	1.76	0.69	< 0.001*	InfRes, ImmSysPro
Interleukin-17D (IL17D)	Q8TAD2	0.00 (0.00–8.38)	9.80 (0.00–22.84)	12.66	2.01	0.79	0.002*	InfRes
Interleukin-18 (IL18)	Q14116	23.70 (1.15–243.51)	165.22 (29.32–1145.19)	0.68	1.57	0.62	< 0.001*	InfRes, ImmSysPro, RegTissRem, PosRegTisRem
Lymphotoxin-alpha, TNF-β (LTA)	P01374	0.08 (0.00–15)	0.13 (0.02–.23)	0.14	1.18	0.46	0.574	ImmSysPro, RegCelDet
Macrophage migration inhibitory factor (MIF)	P14174	1284.84 (384.19–19792.04)	5110.70 (846.04–20022.03)	1.61	1.74	0.68	< 0.001*	InfRes, ImmSysPro, RegCelDet
Macrophage colony-stimulating factor 1, (CSF-1)	P09603	0.18 (0.00–92)	0.46 (0.14–1.22)	0.10	1.66	0.65	< 0.001*	InfRes, ImmSysPro, RegCelDet
Thrombopoietin, (THPO)	P40225	0.00 (0.00–1.95)	0.76 (0.00–3.79)	2.09	1.45	0.57	0.55	ImmSysPro
Vascular endothelial growth factor A (VEGFA)	P15692	10.77 (2.90–49.08)	22.48 (4.85–54.01)	0.80	1.12	0.44	0.15	ImmSysPro, RegCelDet, VasWouHel

Protein association number according to Uniprot. Data are expressed as absolute number (pg/ml) or median and minimum/maximum number. VIP > 1.0 and p(corr) > 0.4 were considered as significant. Mann-Whitney U test was conducted in SPSS to test significance level of comparison between the baseline and control measurements and directly after removal of the NIV mask. A p-value < 0.05 was considered as significant.

*InfRes* inflammatory response, *ImmSysPro* immune system process, *RegTissRem* regulation of tissue remodeling, *PosRegTisRem* positive regulation of tissue remodeling, *RegCelDet* regulation of cell death, *VasWouHel* vascular wound healing.

**Figure 5:**
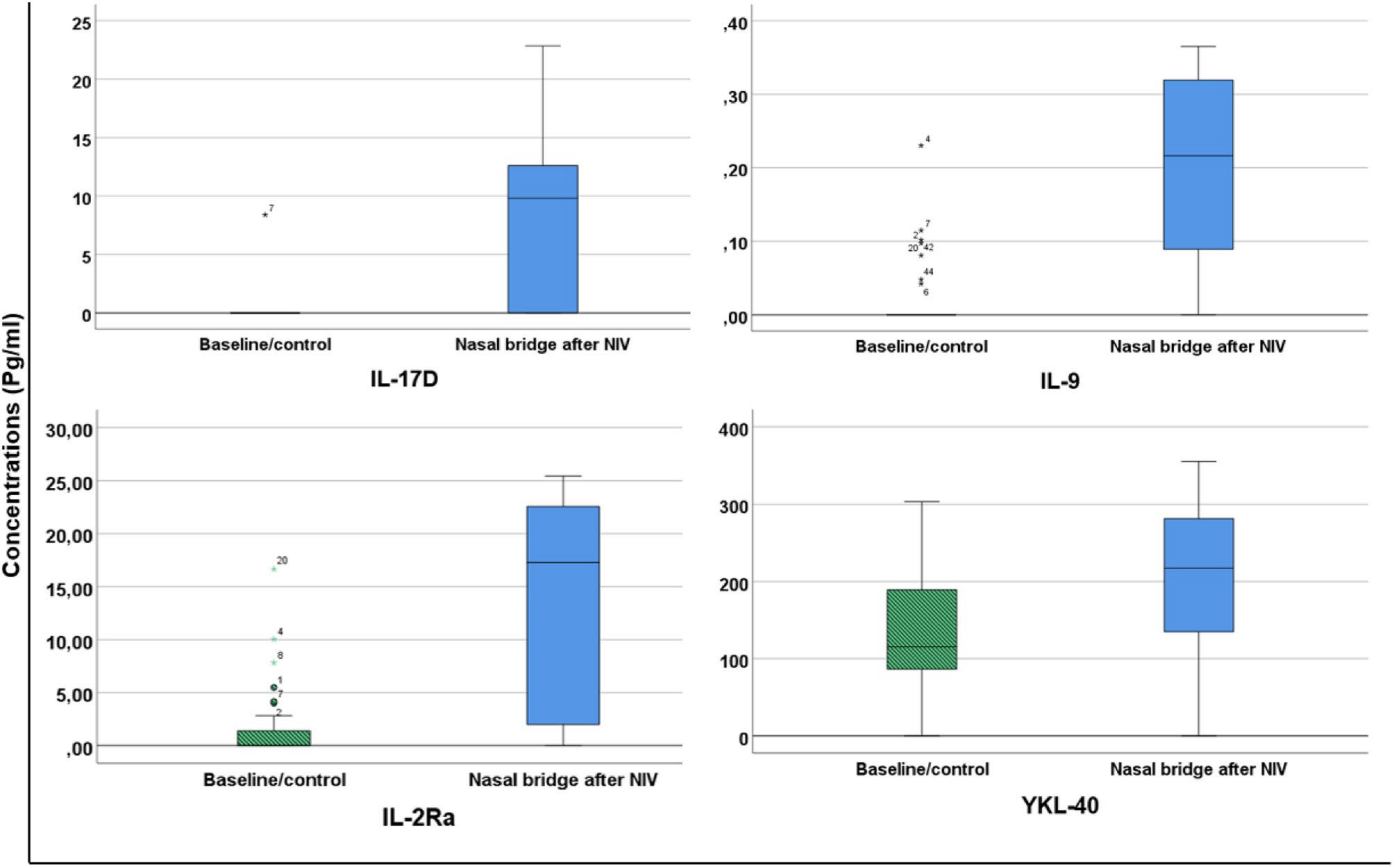
Boxplots showing variations in concentrations of inflammatory proteins between baseline/control and after increased loading in skin. Tested using Mann–Whitney U-test. Median levels are represented by horizontal lines. Green/striped boxplots represent baseline/control measurement, blue boxplots represent measurement directly after removal of NIV-mask. IL-17D (p-value = 0.002), IL-9 (p-value = < 0.001), IL-2Ra (p-value = 0.001), YKL-40 (p-value = 0.098). *NIV*  noninvasive therapy, *IL-17D* interleukin 17D, *IL-9* interleukin 9, *IL-2Ra* interleukin-2 receptor subunit alpha, *YKL-40* chitinase-3-like protein 1.

From the OPLS-DA model, the 21 inflammatory proteins most important for separation between groups was tested using Mann-Whitney U-test to test differences between baseline/control site and nasal bridge after NIV-therapy. The concentration of each protein was compared between baseline/control site and after loading (directly after removal of the NIV-mask). The 4 proteins with the highest absolute p(corr) are presented as boxplots in Fig. 5 (IL-17D, IL-9, IL-2Ra & YKL-40). There was a statistically significant increase after loading in IL2RA (p-value = 0.001), IL17D (p-value = 0.002) and in IL-9 (p-value = < 0.001). There was no significant increase in YKL-40 (p-value = 0.098), but the increase after loading was twofold. There was a large inter-subject variability in the protein concentration, and at baseline the concentrations in some proteins were very low (IL-17D, IL-9). Boxplots of all 21 proteins are available as supplementary file (Supplementary Figure).

### Bioinformatics

**Figure 6:**
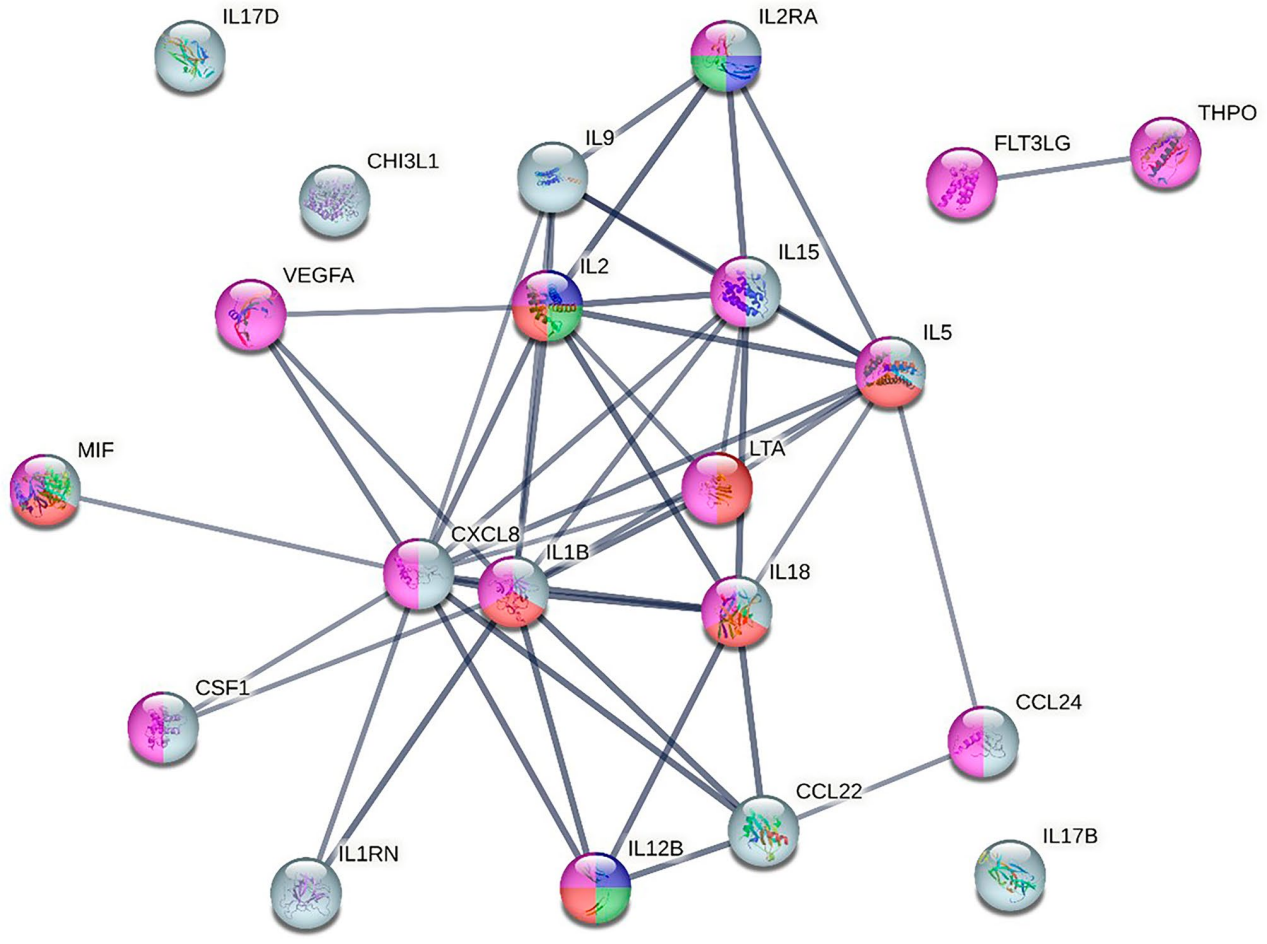
Pathway analysis of the 21 proteins most important for the separation before and after NIV therapy using the STRING database. Inflammatory pathways highlighted: grey = inflammatory response, purple = positive regulation of immune system process, green = negative regulation of inflammatory response, blue = negative regulation of immune response, red = positive regulation of immune effector process. High confidence interval (0.700), PPI enrichment value < 0.001.

The 21 biomarkers that contributed to the discrimination between increased loading and control site were analysed to investigate the association between the proteins and the activated pathway (Fig. 6*).* PPI enrichment in the STRING network was < 0.001, confidence interval 0.70. The proteins of interest were involved in the biological process of inflammatory actions, namely, inflammatory response, positive regulation of immune system process, negative regulation of inflammatory response, negative regulation of immune response and positive regulation of immune effector process.

**Figure 7:**
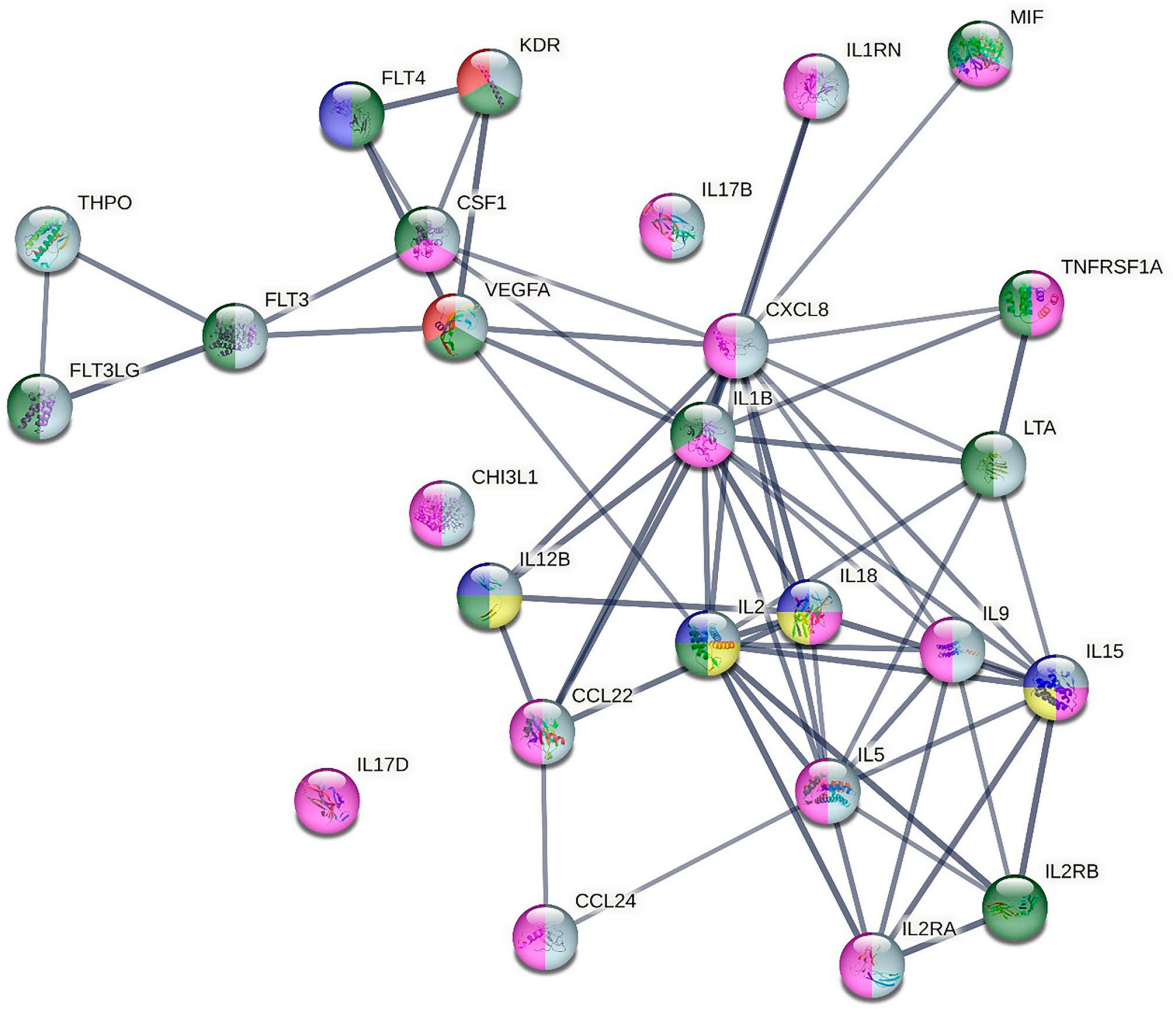
Extended pathway analysis of proteins showing their biological processes. Pathways highlighted: purple = inflammatory response, grey = immune system process, green = regulation of cell death, red = vascular wound healing, blue = regulation of tissue remodeling, yellow = positive regulation of tissue remodeling. PPI enrichment was < 0.001.

When adding associated proteins to see larger connections in an extended pathway analysis, further interactions were found. Tumor necrosis factor receptor superfamily member 1A (TNFRSF1A), Interleukin-2 receptor subunit beta (IL2RB), Receptor-type tyrosine-protein kinase (FLT3), Vascular endothelial growth factor receptor 3 (FLT4), and Vascular endothelial growth factor receptor 2 (KDR) were included in this larger protein network, Fig. 7*.* The proteins of interest were involved in the biological process of inflammatory actions, namely, inflammatory response, immune system process, regulation of cell death, vascular wound healing, regulation of tissue remodeling and positive regulation of tissue remodeling.

## Discussion

In this study we explored the inflammatory profile of vulnerable skin sites following non-invasive ventilation (NIV) mask application. For the first time, 60 inflammatory proteins were simultaneously explored after mechanical loading in skin, and we concluded that 21 cytokines and chemokines contributed most to the separation between control group and mechanically loaded skin site over the nasal bridge. Further, 14 cytokines and chemokines were statistically significant in their elevation between control site and mechanical loading, implying potential as biomarkers for early staged tissue damage. The extended network analysis showed that these cytokines and chemokines may represent the precursor to skin damage since they were pro-inflammatory (Interleukin-1 beta (IL-1β), p-value 0.013), non-inflammatory (IL-1receptor antagonist (IL-1Ra), p-value =  < 0.001), regulators of tissue remodeling (Interleukin-18 (IL-18), p-value < 0.001) and regulators of cell death (Macrophage colony-stimulating factor 1 (M-CSF) (p-value < 0.001).

The inflammatory response where present immediately after removal of the NIV mask and the pro-inflammatory cytokines IL-1beta (IL-1β) and the early inflammatory chemokine Interleukin-8 (IL-8) were parts of the observed protein pathway. Skin keratinocytes have the mechanisms to both initiate and reduce inflammation since they produce the pro-inflammatory cytokines IL-1alpha (IL-1α) and IL-1beta (IL-1β) and the Interleukin-1antagonist (IL-1Ra)^27^. Similarly, IL-8 recruits the first wave of inflammatory cells^28^ and has shown promising results as biomarkers for early staged pressure ulcers^21,29^. In this present study, median values of IL-8 showed a trend of difference between baseline/control site and the nasal bridge after NIV-therapy (p = 0.07). Furthermore, an increase in IL-8 after one hour of 150 mmHg loading was shown in vitro^30^, similar to pressures values obtained using NIV-masks (158 ± 54 mmHg)^10^. In this present study, the duration of NIV-therapy was shorter (mean 22 min) compared to the study of Cornelissen et al.^30^, which may explain that IL-8 were not statically significant between control and nasal bridge after NIV-therapy but were important for the protein network.

The present study observed that IL-1α was not part of the 21 proteins that contributed most to the separation between increased loading and control site, which was contradictory to previous research which has identified IL-1α as a potential biomarker for early skin tissue damage^20,31^. Indeed, the ratio between IL-1α and IL-1Ra have been identified as a sensitive and specific marker for healthy and stage 1 PU skin sites^21^. Nevertheless, IL-1β were an important part of the network and contributed significant to the differences between control and after loading (p-value 0.013). IL-1α is an alarmin, fast proinflammatory cytokine^32^ that increases as a response from several stimuli, including chemical insults, which may have limited specificity to skin damage from mechanical loading^33^. IL-1β is not an alarmin and requires activation in several steps to be active^32,34,35^ that may make it more specific to local mechanically induced skin damage.

Tissue remodeling and vascular wound healing were important biological processes in the extended protein network which were unexpected since the measurement was done directly after removal of the NIV mask after a relatively short period of loading (mean 22 ± 8 min). In clinical routine, depending on the patient’s respiratory status NIV-therapy could be used for several hours to improve the respiratory status. Interleukin-15 (IL-15) and Interleukin 18 (IL-18) regulates tissue remodeling and were important for the separation between loading site and control. They were both significantly increased (IL-15 p-value 0.009, IL-18 p-value < 0.001) despite the relatively short duration of loading and the timepoint of measurement directly after removal of the NIV-mask. This study explored the inflammatory tissue response prior wound development. However, 64% (n = 7) of the study population had developed a visible erythema at the loading site by removal of the NIV-mask which may be caused by hyperemia and vasodilation after increased loading in skin^36^. This highlights the importance of further assessing the period after load to further explore the time-resolved interactions between different biomarkers in the tissue recovery phase or process of wound development in the tissue.

In the extended protein network analysis in this study, Vascular endothelial growth factor receptor 2 (KDR) and vascular endothelial growth factor alpha (VEGF) were important in the biological process of regulation of cell death and vascular wound healing. KDR facilitates the chemotactic activities of vascular endothelial growth factor (VEGF), which induces endothelial cell growth and vascular permeability as a response to ischemia and hypoxia^37^. This may imply that skin cells under this short duration of mechanical loading were deformed sufficiently to occlude microvascular circulation. Indeed, the corresponding loads ~ 100 mmHg at the bridge of the nose has previously been shown to be sufficient to cause localized ischemia^11,38,39^. However, above from the previously mentioned chemokines, Macrophage colony-stimulating factor 1 (CSF1) and Macrophage migration inhibitory factor (MIF) were important in the biological process of regulation of cell death. Their mean values between loaded site vs control site were significantly increased between the sites (CSF1 p-value < 0.001, MIF p-value < 0.001). CSF1 increases with inflammatory stimuli and promotes tissue repair^40^ and MIF increases in response to cell death and can regulate other cytokines such as Interleukin-2 (IL-2), IL-6, IL-1 β and IL-18^41^ which all were important inflammatory proteins in this study.

There are some methodological limitations in this study. Firstly, the use of Sebutape is validated and safe but this method could only collect proteins that are released from skin surface and thereby may not reflect deeper tissue physiology. Secondly, due to the explorative nature of the study including a small study population, no generalizations of the results are possible. Further, the patient population had increased inflammatory levels in blood that may have influenced the local inflammatory response. However, the study design mitigated the effect of systemic inflammation by using a local control site and time dependent changes following loading. This may indicate that the systemic inflammation may not be affecting the local response, in contrast to previous studies^42^. Finally, the mechanical loading in skin by NIV-therapy had a range between 9 to 80 min (mean 22 min), and the duration of mechanical loading most certainly influences the inflammatory response in skin. The impact of the duration of mechanical loading is important to further explore in future studies since this observational study with ordinary treatment regimens reflects the real-life situations with clinical application and use of NIV-masks. In summary, this study contributed with new insights into the complexity of early inflammation after mechanical loading in skin.

This study contributes with new knowledge in the complexity of early inflammation in vulnerable skin sites following NIV-mask application. In practice there is little standardization in application of NIV-masks and preventive measures to avoid tissue damage from NIV-masks, which develops in 50% of patients receiving the therapy for 2 hours^7^. Using a multiplex immunoassay technology with a broad panel of 71 inflammatory proteins this study adds new insights to pressure ulcer formation. The extended pathway analysis showed biological processes involved in cell death, tissue remodeling and vascular wound healing directly after removal of the face mask. Further, 14 cytokines and chemokines had a significant differentiation between control site and loaded site and may be potential biomarkers for early skin tissue damage. Further studies are needed for validation in a larger study population and to explore the expression and interaction of the biomarkers in the tissue recovery period after loading, with the potential of creating point of care biosensors for the early detection of skin damage.

### Supplementary Information

Supplementary Figure.

## Data Availability

All relevant data are included in the manuscript, tables, and figures. Additional data will be provided from the corresponding author upon reasonable request.
