# Supplementary figures and images for: The use of a protein network analysis to explore the complexity of early skin inflammation after oronasal mask application- A pilot study
Source: Sci Rep. 2024 Jul 19;14:16691. doi: 10.1038/s41598-024-67583-9 (PMC11271608; doi:10.1038/s41598-024-67583-9)

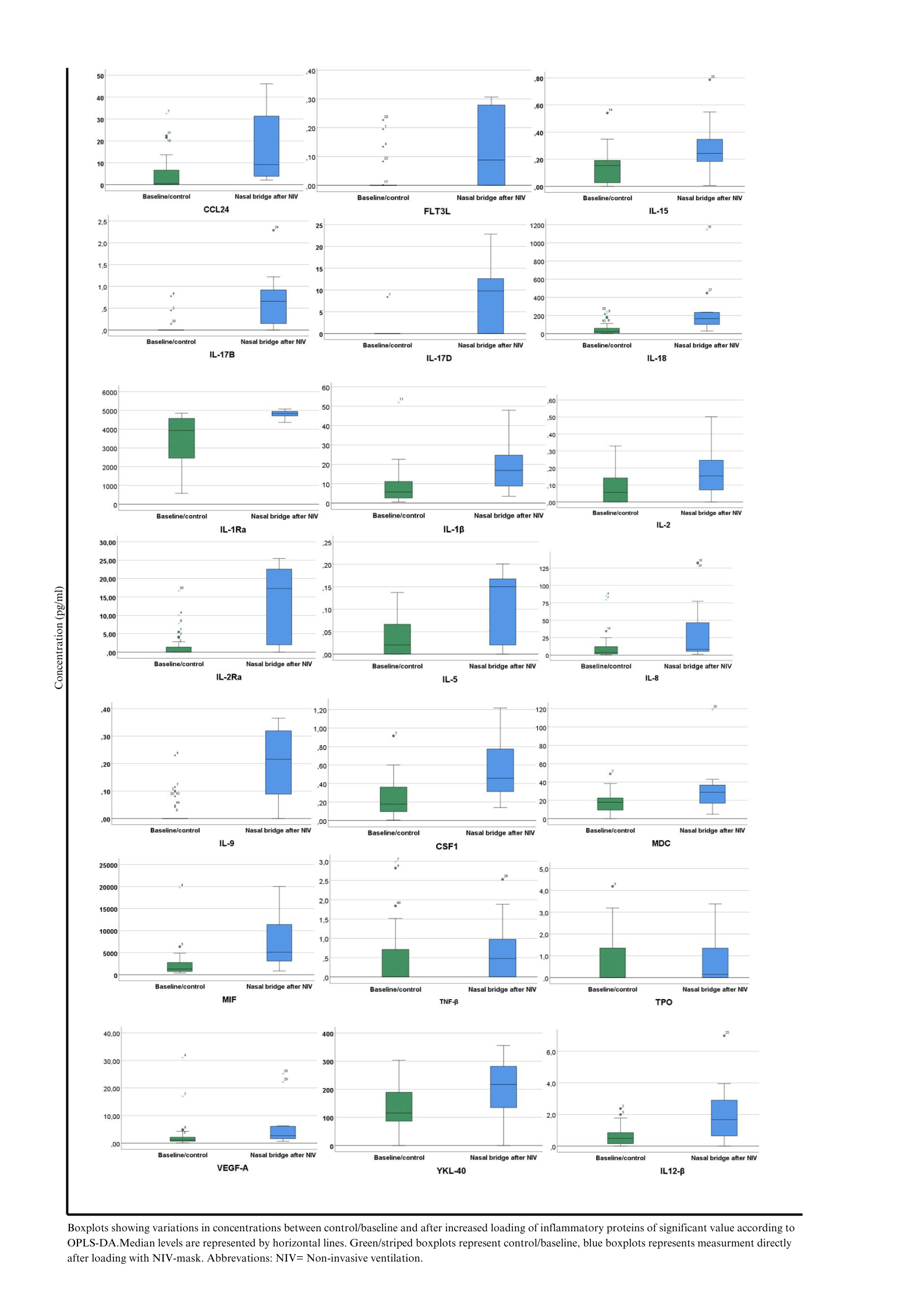

Supplement: Supplementary file 1 — Supplementary Figure. [file 41598_2024_67583_MOESM1_ESM.png]
